# Periostin suppression induces decorin secretion leading to reduced breast cancer cell motility and invasion

**DOI:** 10.1038/srep07069

**Published:** 2014-11-17

**Authors:** Toshiyuki Ishiba, Makoto Nagahara, Tsuyoshi Nakagawa, Takanobu Sato, Toshiaki Ishikawa, Hiroyuki Uetake, Kenichi Sugihara, Yoshio Miki, Akira Nakanishi

**Affiliations:** 1 Department of Molecular Genetics, Medical Research Institute, Tokyo Medical and Dental University (TMDU); 2 Department of Surgical Oncology, Tokyo Medical and Dental University (TMDU); 3 Department of Molecular Diagnosis, Cancer Institute, The Japanese Foundation of Cancer Research (JFCR)

## Abstract

The ability of cancer cells to metastasize is dependent on the interactions between their cell-surface molecules and the microenvironment. However, the tumor microenvironment, especially the cancer-associated stroma, is poorly understood. To identify proteins present in the stroma, we focused on phyllodes tumors, rare breast tumors that contain breast stromal cells. We compared the expression of proteins between phyllodes tumor and normal tissues using an iTRAQ-based quantitative proteomic approach. Decorin was expressed at reduced levels in phyllodes tumor tissues, whereas periostin was upregulated; this result was validated by immunohistochemical analysis of phyllodes tumors from 35 patients. Additionally, by immunoprecipitation and mass spectrometry, we confirmed that decorin forms a complex with periostin in both phyllodes tumors and BT-20 breast cancer cells. Following siRNA-mediated knockdown of periostin in T-47D cells, secreted decorin in the culture medium could be detected by multiple reaction monitoring (MRM). Furthermore, periostin knockdown in BT-20 cells and overexpression of decorin in MDA-MB-231 cells inhibited cell motility and invasion. Our results reveal the molecular details of the periostin–decorin complex in both phyllodes tumor tissues and breast cancer cells; this interaction may represent a novel target for anti-cancer therapy.

The tumor microenvironment plays a critical role in cancer progression. The stromal and epithelial cells that constitute the tumor microenvironment strongly influence tumor proliferation, invasion, and metastasis, and the phenotypes of tumors are largely determined by interactions between cancer cells and their microenvironment^1^,^2^,^3^. Analyses of cancerous stroma are crucial to improving our understanding of cancer.

Recent studies have shown that periostin and decorin are components of the extracellular matrix that affect the biology of various types of cancer^4^,^5^. Periostin, also known as OSF-2, is a 93-kDa matrix N-glycoprotein. Upregulation of periostin has been observed in many human tumors, including cancers of the lung^6^,^7^, colon^8^, skin^9^, pancreas^10^, thyroid^11^, ovary^12^, breast^13^, and prostate^14^; periostin overexpression is associated with increased tumor invasion and accelerated progression^15^,^16^. Furthermore, high stromal periostin expression is a prognostic factor associated with reduced progression-free survival^12^. Gillan *et al.* reported that periostin interacts with integrin receptors^17^. Purified recombinant periostin supported the attachment of human ovarian surface epithelia (HOSE) and human ovarian carcinoma cells (Sk-ov-3). Sk-ov-3 cells express the β1, αVβ3, and αVβ5 integrins. Attachment of Sk-ov-3 cells to a periostin-coated plate was inhibited by anti-αVβ3 or anti-αVβ5 antibody, whereas function-blocking antibodies against β1 integrins inhibited the attachment of Sk-ov-3 cells to fibronectin. On the other hand, periostin overexpressed in cancer-associated fibroblasts (CAFs) is a key component of primary tumor niche and supports cancer cell proliferation^18^; likewise, in colon cancer, periostin secreted by CAFs supports the growth of epithelial components^19^.

Small leucine-rich proteoglycans (SLRPs) are components of the extracellular matrix, which is altered in the environment surrounding a tumor. SLRPs such as decorin, lumican, and biglycan are expressed in the vicinity of colon, pancreas, breast, and prostate cancers^20^,^21^,^22^. Decorin is a proteoglycan, on average 90–140 kDa in molecular weight, consisting of a 40-kDa protein core containing leucine repeats conjugated to a glycosaminoglycan chain consisting of either chondroitin sulfate or dermatan sulfate. Relative to adjacent normal stroma, decorin expression is downregulated in fibroblast-like cells within the stroma surrounding human breast tumors^20^. Furthermore, decorin-expressing tumor xenografts grow at significantly lower rates and exhibit significantly suppressed neovascularization^23^. Decorin binds collagen I, regulates fibrillogenesis^24^,^25^, and protects collagen fibrils from proteolytic cleavage by various collagenases^26^.

Decorin has recently emerged as a potential natural anticancer agent produced by normal cells^27^. Specifically, decorin neutralizes the bioactivity of transforming growth factor–beta1 (TGF-β1), an autocrine factor that stimulates the growth of cancer cells^28^,^29^. Collectively, the set of proteins that interact with decorin (the ‘interactome') generates a powerful antitumorigenic signal by potently repressing tumor cell proliferation, survival, migration, and angiogenesis^30^. Fibroblasts secrete several components of the extracellular matrix, including decorin^31^,^32^, and also play important roles in influencing progression toward malignancy^33^. Therefore, fibroblasts are key determinants of the malignant progression of cancer, and thus represent an important target for cancer therapies^34^.

In this study, we focused on phyllodes tumors, which are composed of epithelial and cellular stromal components of the breast. We compared tissue-specific protein expression in phyllodes tumor and normal tissues by iTRAQ (isobaric tag for relative and absolute quantitation) and tandem mass spectroscopy. These analyses revealed that decorin was expressed at lower levels, whereas periostin expression was upregulated, in phyllodes tumor tissues and cancer cells. Furthermore, we characterized the periostin–decorin complex. In particular, we found that knockdown of periostin results in translocation of decorin from the cytoplasm to the extracellular space, leading to the inhibition of cancer cell migration and invasion.

## Results

### Periostin upregulation and decorin downregulation in phyllodes tumor tissue

**Figure 1:**
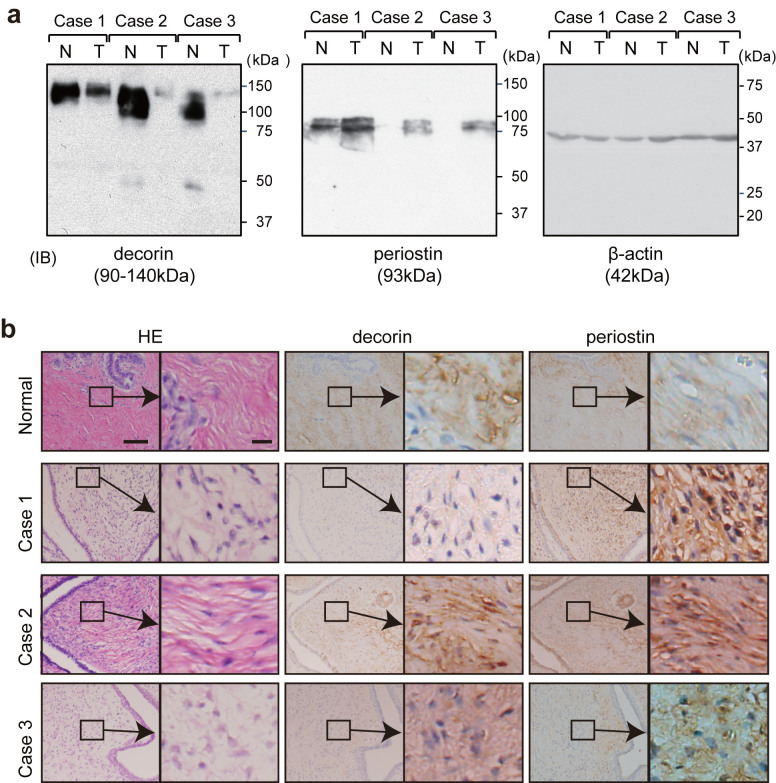
Decorin and periostin expression levels in normal and tumor tissues. (a) Tissue lysates were immunoblotted with anti-decorin and anti-periostin antibodies. N, normal tissue; T, tumor tissue. (b) Tumor and normal tissues were fixed with formalin. Serial sections were visualized by hematoxylin and eosin staining (HE) and immunohistochemical staining with anti-decorin and anti-periostin antibodies. Bar = 100 μm. Insets indicate magnified views in right panel. Bar = 20 μm.

Cancer stroma consists mainly of cancer-associated fibroblasts (CAFs), which affect aspects of the tumor microenvironment such as angiogenesis, invasion, and metastasis. CAFs promote tumor progression in breast cancer, but the details of their role remain unclear, primarily because the collection of CAFs from cancer tissue is technically difficult. Therefore, in this study we focused on phyllodes tumors, which consist of breast stromal and epithelial cells. We used the iTRAQ-based quantitative proteomic approach to identify proteins that were differentially expressed between phyllodes tumor and normal tissues. As shown in Supplementary Figure S1, a total of 2041 proteins in case 1, 2338 proteins in case 2, and 4281 proteins in case 3 were identified by ProteinPilot. Of the identified proteins, 99.9% in case 1, 99.8% in case 2, and 99.6% in case 3 were labeled with iTRAQ tags. Next, we selected proteins that were at least 3-fold more abundant in one of these tissue types (tag 114/tag 117 > 3 for proteins enriched in normal tissue, or tag 117/tag 114 > 3 for proteins enriched in tumor tissue). We set a cutoff of 3-fold according to the method described by Juling Ji et al^35^. A total of 101 proteins were detected multiple times in three serial measurements from the same KCl concentration fractions (Supplementary Table S1). Finally, from among the proteins detected in all three cases, we selected five proteins enriched in normal tissues and two proteins in phyllodes tumor tissues. Decorin, mimecan, hemoglobin subunit alpha, hemoglobin subunit beta, and keratin type I cytoskeletal 19 were upregulated in normal tissue, whereas periostin and versican core protein were upregulated in phyllodes tumor tissue (Supplementary Figure S1). Periostin and decorin are components of the extracellular matrix. Periostin upregulation has been reported in many types of cancer, and it is consequently defined as a tumor-enhancing factor^10^,^17^,^36^,^37^. On the other hand, decorin upregulation inhibits tumor growth by antagonizing tumor angiogenesis^30^. Both proteins have recently been discussed as potential targets for stroma-targeted anticancer therapy^17^,^30^. Accordingly, we focused our subsequent analyses on decorin and periostin. In all three cases, decorin was expressed at higher levels in normal tissues than in phyllodes tumors, whereas periostin was upregulated in phyllodes tumor, as confirmed by immunoblot analysis (Figure 1a). Immunohistochemistry revealed that decorin and periostin were localized in the extracellular matrix (Figure 1b).

### Decorin is upregulated in normal tissue, and periostin is upregulated in phyllodes tumor tissue, from cancer patients

**Figure 2:**
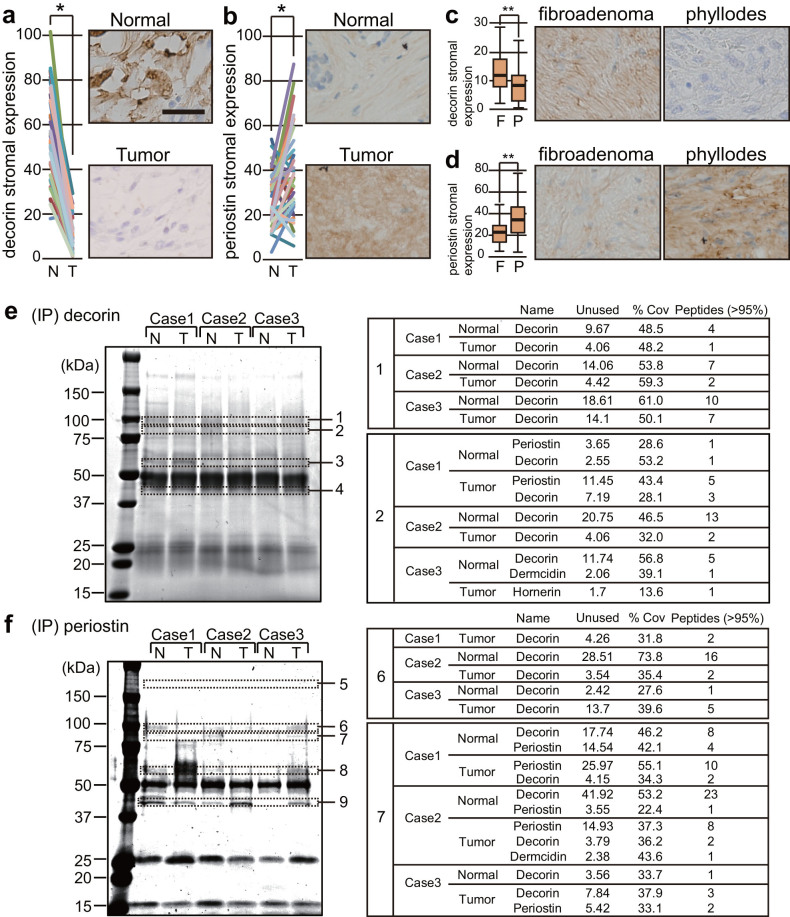
Comparison of stromal decorin and periostin expression levels and complex formation of both proteins in phyllodes tissues. (a, b) Normal tissues (N) and tumor tissues (T) from 35 phyllodes tumor patients were analyzed by immunohistochemical staining. Each colored line connects data points derived from one patient. (c, d) Stromal decorin and periostin expression levels in phyllodes tumors (P) (n = 35) were compared with those in fibroadenomas (F) (n = 37). The intensities of stromal decorin (a, c) and stromal periostin (b, d) expression were quantitated using the ImageJ software. (a)–(d) Figures alongside the graphs show representative samples from each group. Bar = 100 μm. (a, b) P values were determined using the Wilcoxon signed-rank test; P < 0.05 was considered to represent a statistically significant difference (*). (c, d) P values were determined using the Mann-Whitney U test; P < 0.05 was considered to represent a statistically significant difference (**).(e) Complex between periostin and decorin in normal and tumor tissues from phyllodes tumor patients. Anti-decorin immunoprecipitate in normal tissue (N) or tumor tissue (T) lysate from phyllodes tumor patients was subjected to SDS-PAGE, followed by silver staining. Gel bands representing differences between normal tissue and phyllodes tumor were cut out and subjected to in-gel trypsin digestion and mass spectrometry. Proteins identified from gel bands 1 and 2 are shown to the right of the SDS-PAGE image. Proteins identified from other bands are shown in Supplementary Table S2. (f) Anti-periostin immunoprecipitates in normal tissue (N) or tumor tissue (T) lysate from phyllodes tumor patients were subjected to SDS-PAGE, followed by silver staining. Proteins identified from gel bands 6 and 7 are shown to the right of the SDS-PAGE image. Proteins identified from other bands are shown in Supplementary Table S2.

To validate the accuracy of the results described above, we performed immunohistochemical analysis to examine the levels of decorin and periostin in tumor and normal tissues from 35 phyllodes tumor patients. Decorin expression in normal tissues was higher than in tumor tissues (P < 0.001, Wilcoxon signed-rank test; n = 35) (Figure 2a), whereas periostin expression was lower in normal tissues (P = 0.005, Wilcoxon signed-rank test; n = 35) (Figure 2b). Our data suggests that downregulation of decorin and upregulation of periostin are correlated with malignant progression of tumors.

Next, we verified the expression of both proteins in phyllodes tumors (n = 35) and breast fibroadenomas (n = 37) by immunohistochemical analysis. Fibroadenomas, the most common benign breast tumors, arise from intralobular fibrous tissue. Decorin was present at higher levels in fibroadenomas than in phyllodes tumors (P = 0.009, Mann-Whitney U test; n = 37, 35) (Figure 2c), whereas periostin was present at lower levels in fibroadenomas (P = 0.007, Mann-Whitney U test; n = 37, 35) (Figure 2d). These results suggest that it might be possible to distinguish phyllodes tumors and fibroadenomas by comparing the relative expression levels of decorin and periostin.

### Complex between periostin and decorin in phyllodes tumor tissue

To identify decorin- or periostin-binding proteins in normal and phyllodes tumor tissues, we immunoprecipitated both proteins, and subjected the immunoprecipitates to SDS-PAGE and silver staining. Gel bands representing differences between normal and phyllodes tumor tissue were cut out and subjected to in-gel trypsin digestion and mass spectrometry (Figure 2e and 2f). Thirteen proteins, including periostin, were identified in the anti-decorin immunoprecipitates (Figure 2e and Supplementary Table S2), and twelve proteins, including decorin, were detected in the anti-periostin immunoprecipitates (Figure 2f and Supplementary Table S2). Protein identifications were accepted on the basis of peptide identifications with greater than 95.0% confidence.

### Silencing of periostin by RNA interference induces secretion of decorin from the cell

**Figure 3:**
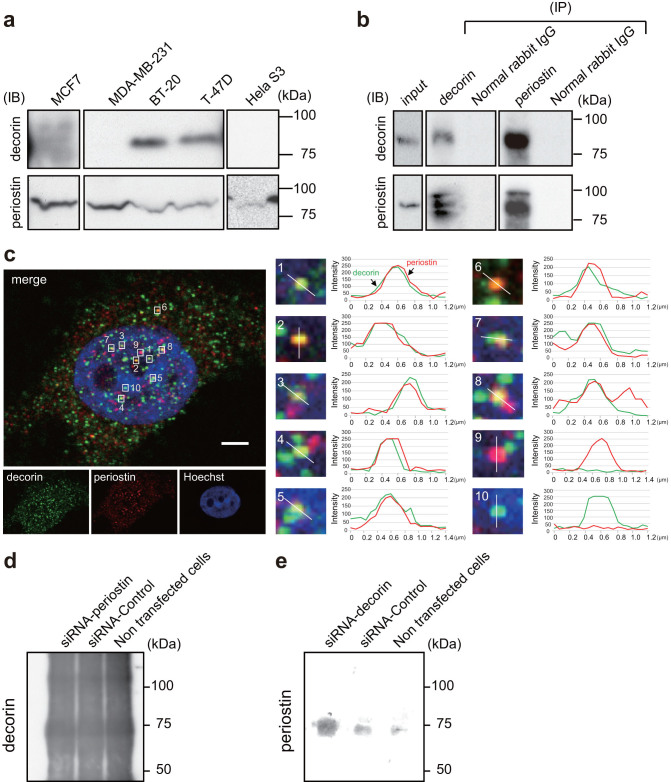
Complex formation between decorin and periostin in BT-20 and T-47D cells. (a) The levels of expression of decorin and periostin in the breast cancer cell lines. Cell lysates from cancer cell lines MCF7, MDA-MB-231, BT-20, T-47D, and HeLa S3 were immunoblotted using anti-decorin and anti-periostin antibodies. Cropped blots are used in the main figures, and full-length blots are included in the supplementary information (Supplementary Figure S2) (b) Anti-periostin or anti-decorin immunoprecipitates from BT-20 cell lysates were subjected to SDS-PAGE, followed by immunoblot analysis with anti-decorin or anti-periostin, respectively. Input lysate was used as a positive control, and normal rabbit IgG was used as a negative control. Cropped blots are used in the main figures, and full-length blots are included in the supplementary information (Supplementary Figure S3) (c) BT-20 cells were fixed, permeabilized, and immunostained with anti-decorin (green) and anti-periostin (red). The cells were observed by high-resolution confocal microscopy on Leica TCS SP8 (left panel). Yellow shows the co-localization of decorin and periostin. Fluorescence intensity profiles along lines were drawn the staining patterns. Bar = 3 μm. Insets indicate magnified views in right panel. Decorin and periostin were closely merged. (d, e) We analyzed secreted decorin or periostin in culture medium of T-47D cells treated with siRNA-periostin (d) or siRNA-decorin (e) by immunoprecipitation, followed by immunoblotting using each antibody.

**Figure 4:**
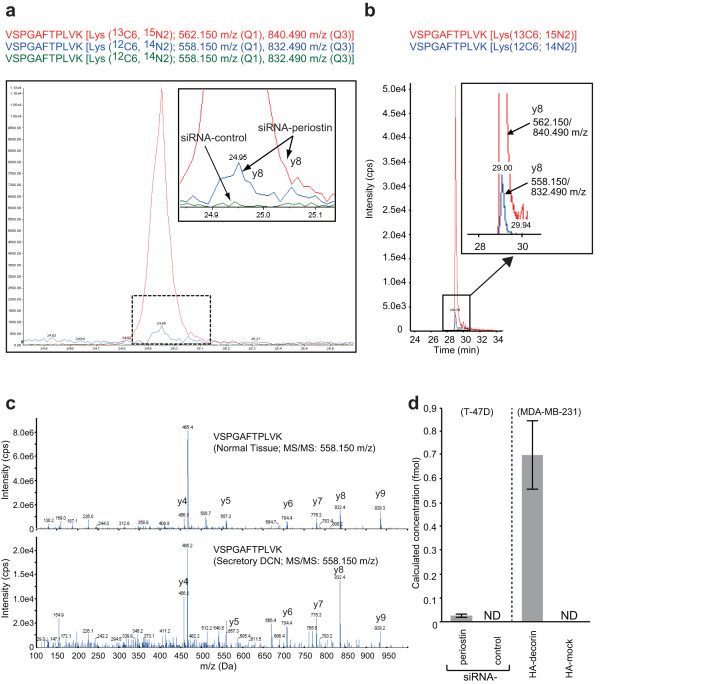
The detection of secreted decorin in the culture medium using multiple reaction monitoring (MRM). (a) MRM chromatograms of VSPGAFTPLVK fragments and their standard (AQUA: red line) analogues. The peptides in culture medium from siRNA-periostin–treated cells (blue line) or siRNA-control–treated cells (green line) were analyzed using the MRM method. The doubly charged precursor mass was chosen as the Q1 mass, and the y8 fragment ion was chosen as the Q3 mass. Insets contain magnified views. (b) MRM chromatograms for VSPGAFTPLVK fragments and their standard (AQUA) analogues. MRM transitions for the endogenous (blue line) and standard (red line) peptides were monitored. For VSPGAFTPLVK, the doubly charged precursor mass was chosen as the Q1 mass, and the y8 fragment ions were chosen as Q3 mass. Insets contain magnified views. (c) MRM-triggered MS/MS product ion spectra obtained by nanoflow LC/MS/MS, comparing normal tissue digested with trypsin (upper) with decorin secreted from decorin-overexpressing MDA-MB-231 cells (lower). The spectrum of the peptide clearly shows y-ion fragments. (d) Calculated concentration of decorin in cell-culture medium of siRNA-periostin–treated T47D cells and decorin-overexpressing MDA-MB-231 cells (n = 3).

We next sought to investigate the functional significance of the interaction between decorin and periostin, both of which are secreted proteins. First, we confirmed that both proteins were expressed in the breast cancer cell lines BT-20 and T-47D; in other cell lines we examined (MCF7, MDA-MB231, and HeLa S3), periostin was present but decorin was not (Figure 3a and Supplementary Figure S2). By co-immunoprecipitation of these proteins from BT-20 lysates, we confirmed that endogenous decorin and periostin interacted in these cells, either directly or indirectly (Figure 3b and Supplementary Figure S3). Immunofluorescence confocal microscopy revealed that decorin and periostin colocalized in BT-20 cells (Figure 3c). Decorin and periostin are components of the extracellular matrix^4^,^5^. We hypothesized that decorin is secreted into the culture medium following treatment with siRNA-periostin. To test this hypothesis, we analyzed secreted decorin in the culture medium by immunoprecipitation, followed by immunoblotting using an anti-decorin antibody, but this antibody was highly cross-reactive and yielded many nonspecific bands(Figure 3d). To overcome this technical obstacle, we used multiple reaction monitoring (MRM) mass spectrometry. In this assay, 5 fmol of standard peptides [VSPGAFTPLVK (^13^C_6_, ^15^N_2_) or DLPPDTTLLDLQNNK (^13^C_6_, ^15^N_2_)] was separated by nano-LC, and the MRM transitions were monitored. The peptides were delivered in 5% (v/v) acetonitrile at a concentration of 5 pmol/μl. Figure 4a and Supplementary Figure S4 show an MRM transition for the co-eluting standard and endogenous peptides (VSPGAFTPLVK and DLPPDTTLLDLQNNK from decorin). In medium in which siRNA-periostin–treated cells were cultured, the spectrum peak corresponding to endogenous peptides overlapped with that of the standard peptide. However, in medium from siRNA-control–treated cells, there was no spectrum peak corresponding to endogenous peptides. On the other hand, periostin was detected by immunoblot analysis with anti-periostin antibody in medium from cells treated with siRNA-decorin cells, but not detected in medium from cells treated with siRNA-control or in non-transfected cells (Figure 3e). Next, we investigated whether MDA-MB-231 cells secrete decorin following decorin transfection, because these cells do not normally express decorin (Figure 3a). Figure 4b and Supplementary Figure S5 show two MRM transitions for the co-eluting standard and endogenous peptide (VSPGAFTPLVK from decorin). The decorin peptide sequence VSPGAFTPLVK was detected using nano-LC-MS/MS, and was determined at a 95% confidence level. We confirmed that the MS/MS spectrum of the peptide derived from secreted decorin was consistent with the decorin spectrum determined in normal tissue. This MRM-based assay demonstrated the high accuracy of target detection by MS/MS analysis (Figure 4c). The absolute quantitations are shown in Figure 4d. We calculated the concentration of decorin in cell-culture medium of siRNA-periostin–treated T47D cells and decorin-overexpressing MDA-MB-231 cells (n = 3). The levels of decorin in medium from siRNA-periostin–treated T47D cells and decorin-overexpressing MDA-MB-231 cells were 0.026 ± 0.007 nM and 0.699 ± 0.143 nM, respectively. The peaks of the endogenous peptide in each control were weak and non-detectable (ND).

### Knockdown of periostin and overexpression of decorin prevent cells motility and invasion

**Figure 5:**
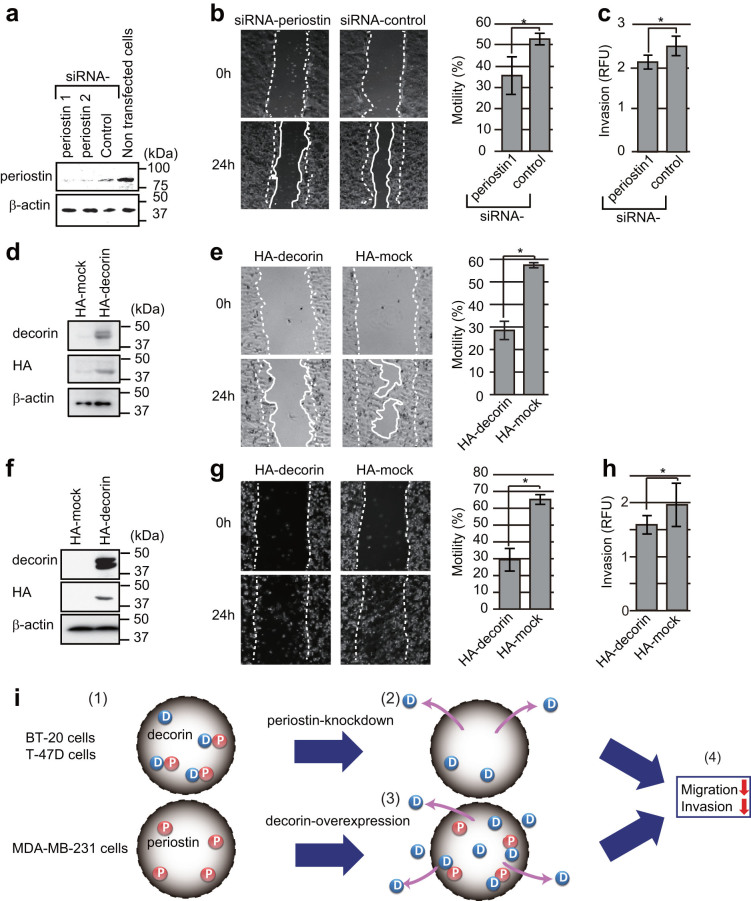
Cell motility and invasion assay following knockdown of periostin or overexpression of decorin. (a) BT-20 cells were treated with siRNA-periostin for 48 h. Each sample was subjected to SDS-PAGE, followed by immunoblot analysis with anti-decorin or anti-periostin. β-actin was used as a loading control. (b) BT-20 cells were treated with siRNA-periostin for 48 h and then evaluated by the wound-healing assay. (c) BT-20 cells were treated with siRNA-periostin for 48 h, and then subjected to the cell invasion assay. (d) BT-20 cells expressing HA-decorin or HA-mock were subjected to SDS-PAGE followed by immunoblot analysis with anti-decorin or anti-HA antibodies. β-actin was used as a loading control. (e) BT-20 cells were transfected with HA-decorin or HA-mock expression vector for 24 h, and then subjected to the wound-healing assay. Column graphs show the means ± SEM of results from three samples. (f) MDA-MB-231 cells expressing HA-decorin or HA-mock were subjected to SDS-PAGE followed by immunoblot analysis with anti-decorin or anti-HA antibodies. β-actin was used as a loading control. (g) MDA-MB-231 cells were transfected with HA-decorin or HA-mock expression vector for 24 h, and then subjected to the wound-healing assay. (h) MDA-MB-231 cells were transfected with a HA-decorin expression vector for 24 h, and then subjected to the cell invasion assay. (b, c, e, g and h) P values were determined using Student's t-test. Asterisks indicate statistically significant differences (P < 0.05). (i) Potential roles of decorin and periostin in phyllodes tumor or breast cancer cell lines (BT-20, T-47D, and MDA-MB-231). (1) Decorin interacts with periostin in phyllodes tumor tissues and BT-20 cells. (2) Secreted decorin is detected in the culture medium of periostin-knockdown T-47D cells. (3) Transient expression of decorin in MDA-MB-231 cells leads to secretion of decorin into the culture medium. (4) Extracellular decorin significantly decreases cell motility and invasion.

The data described above demonstrate that decorin is secreted from periostin-knockdown BT-20 cells, as well as from decorin-overexpressing BT-20 and MDA-MB-231 cells. We next investigated whether these cells could inhibit cell motility and invasion. To this end, we treated BT-20 cells with siRNA-periostin for 48 h. Each sample was subjected to SDS-PAGE, followed by immunoblot analysis with anti-decorin or anti-periostin antibody. β-actin was used as a loading control (Figure 5a). We then compared the proliferation of BT-20 cells treated with siRNA-periostin or siRNA-control. Cell number was measured using the water-soluble tetrazolium-1 (WST-1) assay. Relative fluorescence units (RFU) indicate the relative amount of proliferation. Column graphs show the means ± SEM of results from six samples. Periostin knockdown did not affect cell proliferation, as judged by the WST-1 assay (Supplementary Figure S6). Cell motility was measured in a wound-healing assay by time-lapse microscopy (Figure 5b, 5e, and 5g, left panel). Phase contrast was shown the images at start time (0 h) and 24 h. The dotted lines indicated cells at the start time, and white lines show the tips of migrated cells after 24 h. Bar graphs show the proportion of cell motility and means ± SEM from three samples (Figure 5b, 5e and 5g, right panel). Knockdown of periostin in cells led to a significant decrease in cell motility relative to cells treated with siRNA-control (P = 0.016, Student's T-test; n = 6) (Figure 5b). BT-20 cells transfected with HA-decorin exhibited significantly reduced motility relative to cells transfected with HA-mock vector (P = 0.0003, Student's T-test; n = 3) (Figure 5d and 5e). Similarly, cell invasion was measured using the CytoSelect 96-Well Collagen I Cell Invasion Assay (Cell Biolabs, San Diego, CA, USA). Invasive cells pass through the basement membrane layer, whereas noninvasive cells stay in the upper chamber. After removal of non-invasive cells, invading cells were stained and counted. Column graphs show means ± SEM of results from five samples. Cell invasion was also inhibited by knockdown of periostin (P = 0.016, Student's T-test; n = 4) (Figure 5c). In MDA-MB-231 cells, expression of HA-decorin had no effect on proliferation (Supplementary Figure S7). Expression of HA-decorin in these cells was confirmed by immunoblot analysis (Figure 5f). HA-decorin–expressing cells exhibited significantly reduced motility and invasion relative to cells transfected with HA-mock vector (motility; P = 0.001, Student's T-test; n = 3, invasion; P = 0.032, Student's T-test; n = 6) (Figure 5g and 5h). These data demonstrate that secreted decorin plays a functional role in promoting cancer cell motility and invasion.

## Discussion

In this study, we characterized the interaction between cellular decorin and periostin not only in phyllodes tumors but also in BT-20 breast cancer cells. We detected secreted decorin in the culture medium of periostin-knockdown T-47D cells (Figure 4a and Supplementary Figure S4). Likewise, when decorin was overexpressed in MDA-MB-231 cells, in which decorin is not normally expressed, decorin was detected in the culture medium (Figure 4b and Supplementary Figure S5). These decorin-secreting cells inhibited cell motility and invasion more effectively than control cells (Figure 5).

Our findings demonstrate that periostin is more abundant in phyllodes tumors than in normal tissues (Figure 1, 2a, and 2b), and that it forms a complex with decorin (Figure 2e, 2f, 3b, and 3c). Previous work showed that decorin can delay tumor growth by blocking TGF-β^29^, inhibiting inducers of angiogenesis such as VEGF^23^, or interacting with E-cadherin^38^. On the other hand, knockdown or neutralization of endogenous periostin results in inhibition of cell migration and invasion^39^,^40^, although the mechanism remains unclear. In this report, we describe a novel function of periostin: tethering of free decorin in the cytoplasm of cancer cells, thereby preventing release of decorin to the extracellular space. Our data obtained using both phyllodes tumors and breast cancer cells raise the possibility that knockdown of periostin in cancer cells may cause an effect similar to that of decorin produced by fibroblasts and myofibroblasts. Accordingly, we propose a model in which secretion of decorin is attributed to an inappropriate balance between the levels of decorin and periostin (Figure 5i).

As a component of the extracellular matrix, decorin prevents migration and invasion. Consistent with this, stromal decorin expression adjacent to malignant cells in invasive breast cancer tumors is significantly weaker than that in pure ductal carcinoma in situ (DCIS)^41^. In a previous study of breast cancer^42^, an adenoviral vector containing a decorin transgene retarded primary tumor growth by 67% and greatly reduced pulmonary metastasis. Because secreted decorin inhibits cell motility and invasion, the results of our study suggest the importance of periostin as a potential therapeutic target in cancer cells that express both decorin and periostin. Our mechanistic studies demonstrated that siRNA knockdown of periostin abolishes the interaction with decorin, thereby increasing the level of decorin secreted from cancer cells.

## Methods

Information about immunoblotting analysis, immunoprecipitation, knockdown of gene expression, total RNA extraction, RT-PCR, immunofluorescence and cell proliferation assay can be found in the Supplementary Methods.

### Ethics statement

All human experiments were performed in accordance with the guidelines approved by the Ethics Committee of Tokyo Medical and Dental University (TMDU). The Institutional Review Board of TMDU approved the study, and written informed consent was obtained from each patient before surgery.

### Patients and tissue samples

Tissue specimens analyzed in this study were obtained from 35 patients with phyllodes tumors and 37 patients with fibroadenomas. All patients underwent surgical resection in the Department of Breast Surgery at Tokyo Medical and Dental University, Japan, between March 2003 and August 2012. The clinical characteristics of the tumors we examined are summarized in Table 1. All specimens were formalin-fixed and paraffin-embedded (FFPE). Three tissue samples used for mass spectrometry were snap-frozen in liquid nitrogen and preserved at −80°C.

### Cell lines

BT-20, T-47D, MDA-MB-231, and HeLa S3 cells were generously provided by the Japanese Foundation for Cancer Research. The media for each cell line are summarized in Supplementary Table S3. Cells were maintained at 37°C in a humidified atmosphere containing 5% CO_2_. All cell lines were authenticated in December 2012.

### iTRAQ labeling

Tumor and normal tissues were lysed in T-PER and centrifuged at 100,000 *g* at 4°C for 1 hour. Albumin and IgG were removed from the supernatants using the ProteoSeek™ Albumin/IgG Removal Kit (Thermo Scientific, Waltham, MA, USA), followed by concentration of the sample using Nanosep centrifugal devices (Pall, Ann Arbor, MI, USA). For mass spectrometry, 100 μg of protein lysate was reduced in 25 mM TCEP and 0.05% SDS for 60 min at 60°C, alkylated with methyl methanethiosulfonate for 10 min at RT, and then digested with trypsin at 37°C for 12–16 h. Desalted tryptic peptides were labeled with isobaric tags for relative quantification using iTRAQ reagents (AB SCIEX, Framingham, MA, USA). Briefly, peptides were dried and resuspended in 20 μl iTRAQ dissolution buffer. Trypsin-digested peptides isolated from tumor and normal tissue of three patients with phyllodes tumors were labeled with 2-plex iTRAQ tags; isobaric tags with m/z of 114 were added to normal tissue, and isobaric tags with m/z of 117 were added to tumor tissue (Supplementary Figure S1: cases 1–3). Samples were mixed, passed through a column with elution buffer (KCl: 10, 25, 50, 100, 175, and 350 mM), desalted on MonoSpin C18 columns (GL Science, Tokyo, Japan), and finally prepared for mass spectrometry. Six separate fractions were analyzed by mass spectrometry in at least three trials. The samples were separated by nanoflow liquid chromatography (300 nL/min) on a nano LC Dina-A system (KYA TECH Corp., Tokyo, Japan) in line with a Q-TRAP 5500 instrument (AB SCIEX) using a 75-min gradient of 5–100% acetonitrile in 0.1% formic acid.

### Mass-spectrometry analysis of immunoprecipitates

Samples immunoprecipitated with antibodies against decorin or periostin were subjected to SDS-PAGE, and the gels were stained using the Silver Quest Staining Kit (Invitrogen). The stained gel bands were cut out and treated with dithiothreitol (DTT, Nacalai Tesque, Kyoto, Japan) dissolved in ammonium hydrogen carbonate (Nacalai Tesque), followed by treatment with iodoacetamide (Wako, Osaka, Japan). After the gels were dried, 20 μl of 0.05 pmol/μl trypsin (AB SCIEX) solution was applied to each gel piece and incubated for 12–16 h at 37°C to digest proteins. Digested peptides were extracted by washing the gel pieces twice with 50% trifluoroacetic acid (TFA, Wako), followed by washing with 80% TFA. The purified peptide samples were injected onto a reversed-phase C18 column (HiQ sil C18W-3P, 3 μm, 120 Å; KYA TECH Corp.) and separated by nanoflow liquid chromatography (300 nL/min) on a nano LC Dina-A system (KYA TECH Corp.) in line with a Q-TRAP 5500 instrument (AB SCIEX) using a 75-min gradient of 5–100% acetonitrile in 0.1% formic acid.

### Internal standardization with standard peptides

Standard peptides, VSPGAFTPLVK (^13^C_6_, ^15^N_2_) and DLPPDTTLLDLQNNK (^13^C_6_, ^15^N_2_), were purchased from Thermo Fisher Scientific (Ulm, Germany). The peptides were delivered in 5% (v/v) acetonitrile at a concentration of 5 pmol/μl.

### Multiple reaction monitoring (MRM) analysis

Conditioned medium (CM) was concentrated 100-fold using a Vivaspin 20 (Sartorius Stedim, Göttingen, Germany). The protein concentration in CM was determined using the Bradford protein assay (Bio-Rad, Hercules, CA, USA). Peptides obtained from in-solution digestion of raw CM were analyzed by multiple reaction monitoring (MRM). The doubly charged precursor ion was chosen as the Q1 mass, and the most intense fragment ion from the precursor was chosen as the Q3 mass. The optimized instrument parameters and selected MRM transitions were tested by analyzing the endogenous protein and standard peptides. The samples were separated by nanoflow liquid chromatography (300 nl/min) on a nano LC Dina-A system in line with a Q-TRAP 5500 instrument using a 45-min gradient of 5–100% acetonitrile in 0.1% formic acid. Absolute quantitation was performed using the MultiQuant Software (AB SCIEX). To improve the accuracy of the quantitation, the “heavy” peptide was added into each biological sample at a fixed amount to act as the internal standard for these samples. The actual concentration of peptide in the biological sample was computed from the ratio of the endogenous peptide (light) to the added internal standard (heavy). The concentration C of the targeted endogenous peptide was calculated as 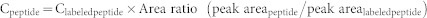


### Immunohistochemical staining

Immunohistochemical staining was carried out by the streptavidin–biotin method using the Histofine SAB-PO kit (Nichirei Co., Tokyo, Japan). Sections (4 μm thick) were cut from each FFPE tissue block. After deparaffinization and rehydration, antigen retrieval treatment was carried out in a temperature-controllable microwave processor (MI-77; Azumaya Co., Tokyo, Japan) at 98°C for 20 min (decorin) or 30 min (periostin) in 10 mM sodium citrate buffer (pH 6.0). Endogenous peroxidase activity was blocked by incubating the sample in a solution of 3% hydrogen peroxide in absolute methanol for 15 min. Nonspecific binding was blocked by treating the slides with 5% EzBlock (including 10% normal goat serum) for 10 min at RT. For detection of decorin, sections were incubated with anti-decorin antibodies (1:1000 dilution), and then beam-irradiated with the MW processor at 27°C for 15 min. The Histofine SAB-PO kit was used for visualization. For detection of periostin, sections were incubated for 90 min at RT with anti-periostin antibodies (1:2000 dilution). The Histofine Simple Stain MAX PO (MULTI) kit (Nichirei Corp.) was used for visualization. Color development was carried out with DAB (0.02% 3,3′-diaminobenzidine tetrahydrochloride; Nichirei Corp) for 10 min at RT. The sections were then counterstained with 1% Mayer's hematoxylin.

### Immunohistochemical evaluation

Immunostaining of decorin and periostin was analyzed under a light microscope. Evaluations of stromal decorin and periostin expression were performed around normal gland and tumor tissue. Digital images were analyzed semiquantitatively. The intensities of decorin and periostin signals were determined using the ImageJ software, according to the method described by Augoff et al^43^. Briefly, random areas at the periphery of lesions were captured as digital images (680 × 512 pixels) with a digital camera. For each digital image, the signal from 10 representative areas was digitized in grayscale ranging from 0 (white) to 255 (black), and these data were used to generate a histogram. Nuclei were omitted from this analysis. Stroma in the negative control samples (i.e., without primary antibody) was used as an internal control. The intensity of the decorin signal was standardized by subtracting the mean intensity of the internal control.

### Wound-healing assay

Confluent cell monolayers were wounded (lightly scratched) with a pipet tip. After careful washing to remove detached cells, the cells were cultured for 24 h. Phase-contrast images were taken every 30 min for 24 h. The width of the wound was monitored using an FW4000-TZ time-lapse microscope (Leica).

### Cell invasion assay

Cell invasion was measured using the CytoSelect 96-Well Collagen I Cell Invasion Assay (Cell Biolabs). Cells (5 × 10^5^) were seeded in serum-free media onto polycarbonate membrane inserts (8 μm thick) whose upper surfaces were coated with a uniform layer of dried Bovine Type I Collagen matrix (Cell Biolabs). Inserts were then submerged in media containing 10% fetal bovine serum (FBS), and the cells were cultured for 24 h. Invading cells were stained with cell stain solution, followed by measurement using a Fluoroskan Ascent plate reader (Thermo Scientific) at 560 nm.

### Statistical analysis

The Wilcoxon signed-rank test was used for comparisons of normal and tumor tissue within the phyllodes patients. Quantitative decorin and periostin stromal expression for comparison of phyllodes and fibroadenoma were analyzed using the Mann-Whitney U test. Student's t-test was used to evaluate the results of proliferation, migration, and invasion assays. P values < 0.05 were considered to indicate statistically significant differences. All statistical analyses were performed using the SPSS software (IBM, Armonk, NY, USA).

## Author Contributions

All the authors contributing to this work have made the following declarations: Y.M., A.N. and T.I. conceived and designed the experiments. A.N. and T.I. performed the experiments. A.N. and T.I. analyzed the data. T.I. contributed reagents and materials tools. Y.M., A.N. and T.I. wrote the manuscript. M.N., T.N., T.S., H.U. and K.S. reviewed the manuscript.

## Supplementary Material

Supplementary Information Supplementary Information

## Figures and Tables

**Table 1:** Clinical characteristics of the tumors. Mean ages of patients with phyllodes tumor and fibroadenoma were 39.3 and 30.1 years, respectively. All patients were female

		phyllodes tumor	fibroadenoma
		n = 35	n = 37
Sex	Female	35	37
	Male	0	0
Age, y	–30	7	19
	31–40	12	13
	41–50	12	5
	51–	4	0
Operation	partial resection	25	36
	mastectomy	11	1
Grade	benign	16	-
	boarderline	14	-
	malignant	5	-
Tumor size(mm)		74.8(±60.5)	37.2(±29.6)
Cutaneous sympton	+	4	1
-	31	36	
Recurrence	+	6	0
	-	29	37
Mortality	dead	0	0
	alive	35	37
