# Supplementary material for: Periostin suppression induces decorin secretion leading to reduced breast cancer cell motility and invasion
Source: Sci Rep. 2014 Nov 17;4:7069. doi: 10.1038/srep07069 (PMC4233340; doi:10.1038/srep07069)
Supplement: Supplementary Information [file srep07069-s1.pdf]

TITLE

Periostin suppression induces decorin secretion leading to reduced breast cancer cell motility and invasion.

Toshiyuki Ishiba[1,2], Makoto Nagahara [2], Tsuyoshi Nakagawa [2], Takanobu Sato [2], Toshiaki Ishikawa [3], Hiroyuki Uetake [3],
Kenichi Sugihara [2], Yoshio Miki [1,4] Akira Nakanishi [1]

[1] Department of Molecular Genetics, Medical Research Institute, Tokyo Medical and Dental University(TMDU),
[2] Department of Surgical Oncology, Tokyo Medical and Dental University(TMDU),
[3] Department of Translational Oncology, Tokyo Medical and Dental University(TMDU),
[4] Department of Molecular Diagnosis, Cancer Institute, The Japanese Foundation of Cancer Research (JFCR),

Supplementary Figure S1

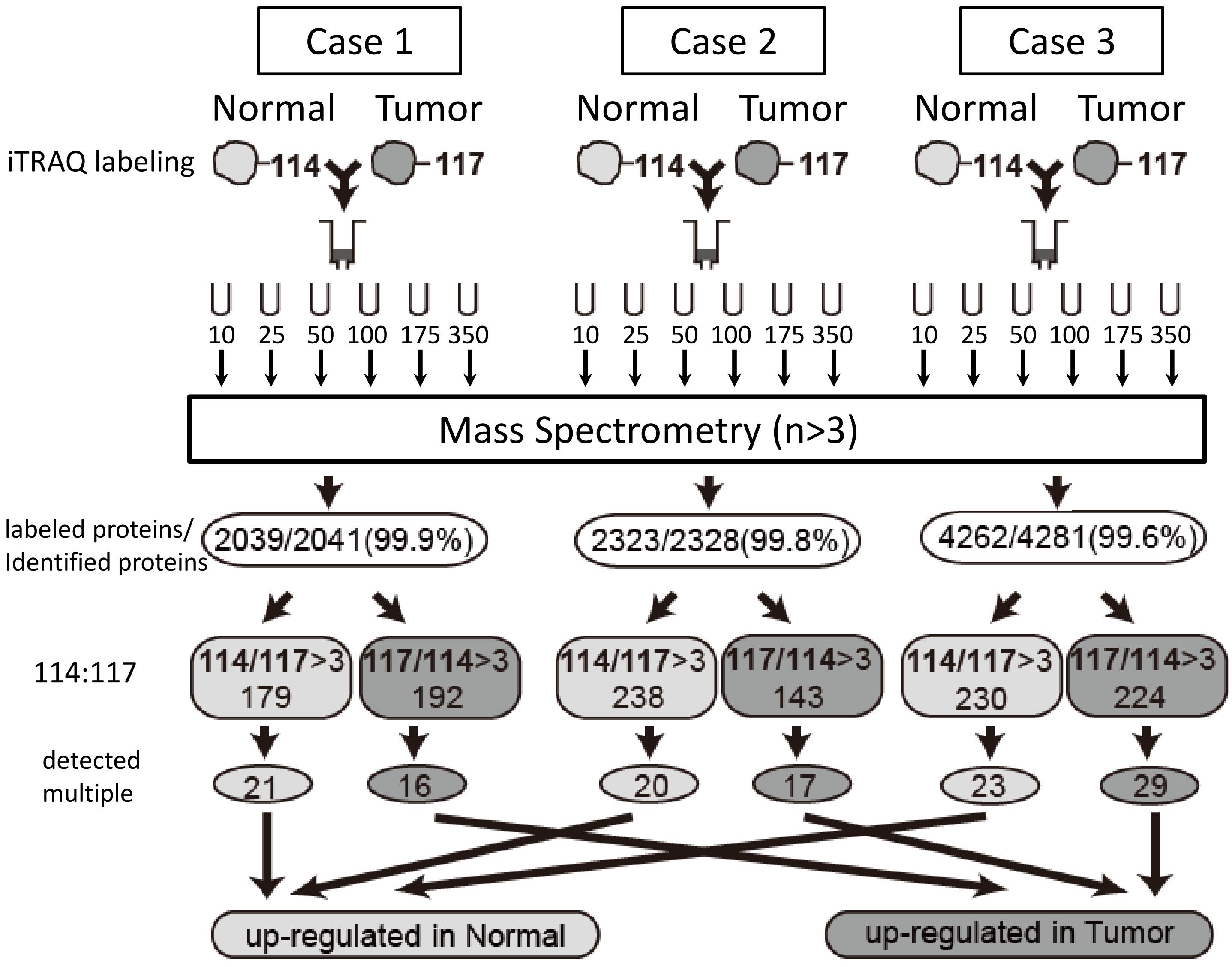

decorin
mimecan
hemoglobin subunit alpha
hemoglobin subunit beta
ketratin, type I cytoskeltal 19

periostin
versican core protein

Supplementary Figure S1. Decorin is upregulated in normal tissues relative to tumor tissues, whereas periostin is upregulated in tumor tissues. Normal and tumor tissues from three phyllodes tumor patients were depleted of albumin and subjected to trypsin digestion. Tryptic peptides were labeled using the iTRAQ reagents. Normal tissue lysates were tagged with isobaric tags with *m/z* of 114, and tumor tissue lysates were tagged with isobaric tags with *m/z* of 117. The tagged lysates were mixed and separated by chromatography on a C_{18} capillary column using elution buffer containing KCl (10, 25, 50, 100, 175, and 350 mM). The six fractions were analyzed by mass spectrometry in at least three trials.

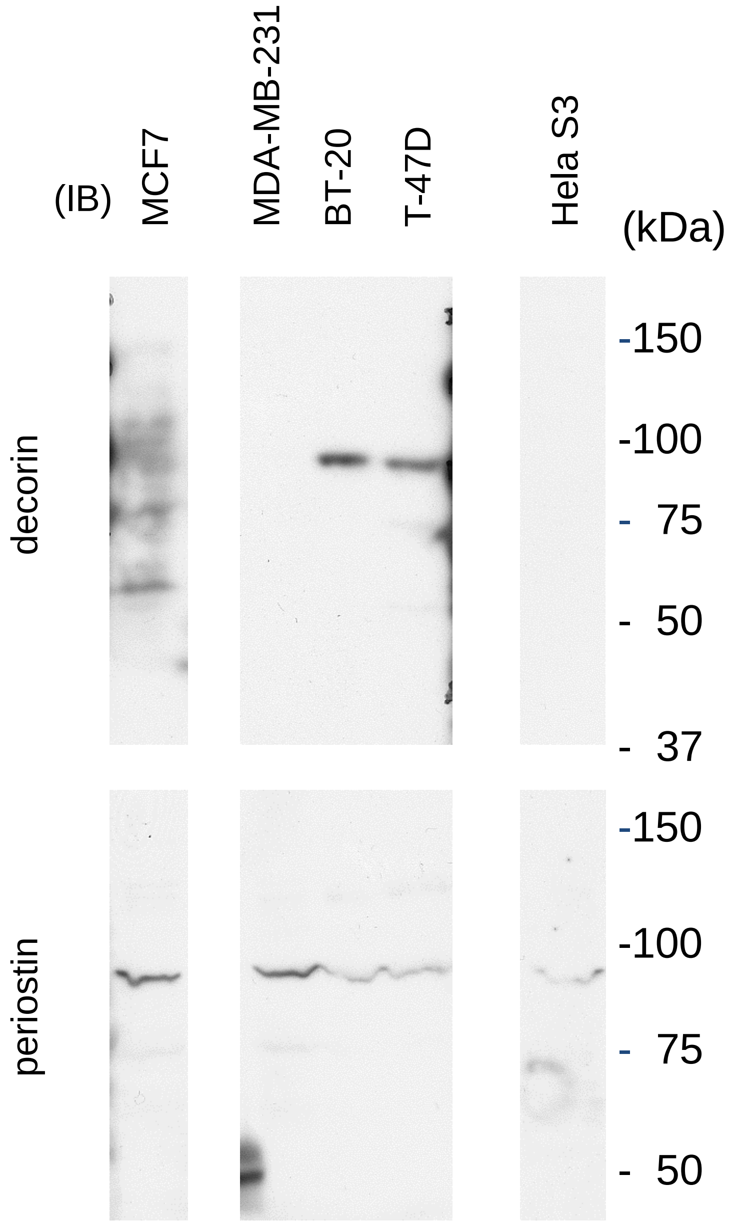

Supplementary Figure S2. The levels of expression of decorin and periostin in the breast cancer cell lines. Cell lysates from cancer cell lines MCF7, MDA-MB-231, BT-20, T-47D and HelaS3 were immunoblotted using anti-decorin and anti-periostin antibodies. These cropped blots are used in the main figure(Figure 3a).

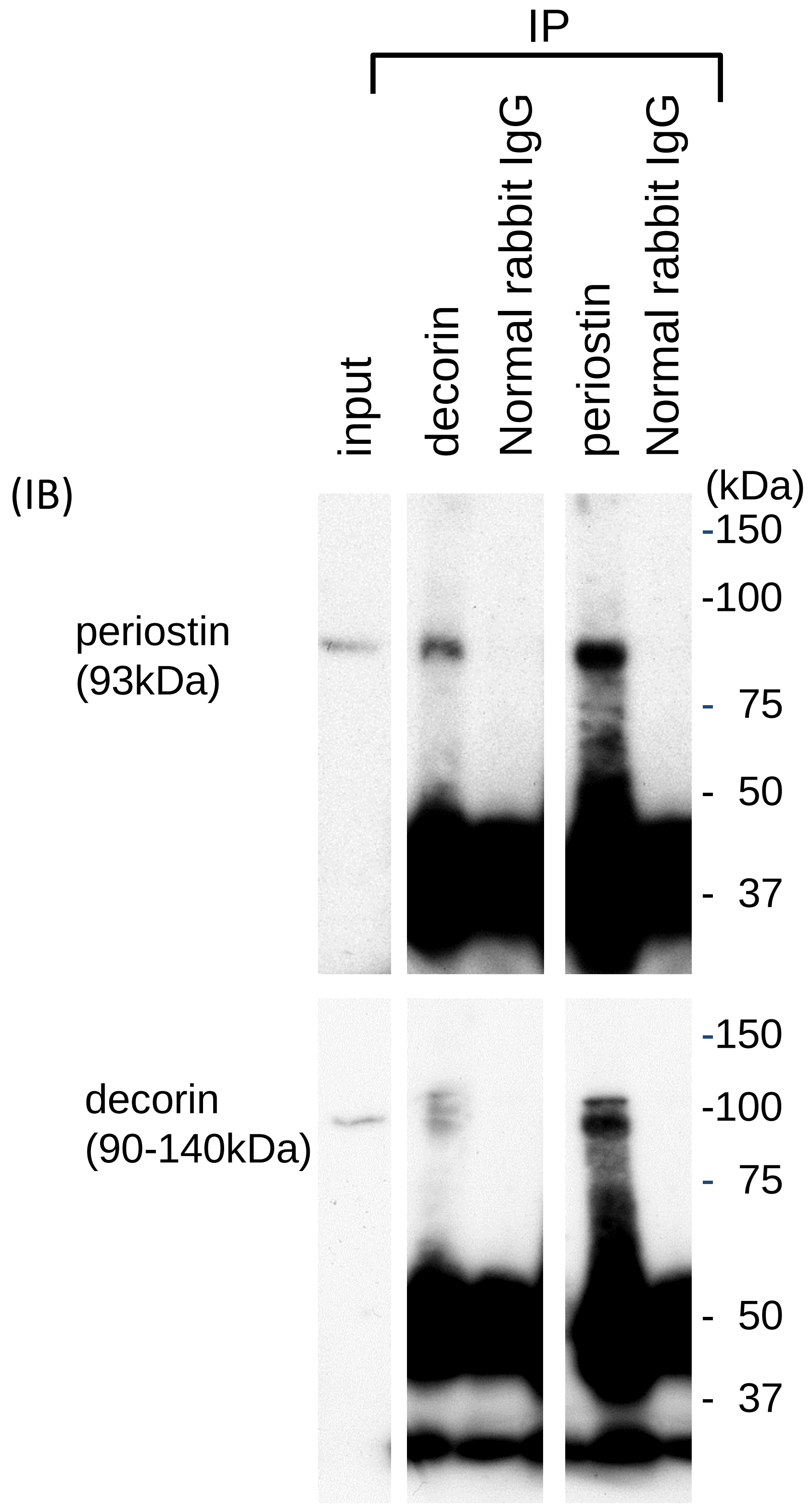

Supplementary Figure S3. Plasma membrane translocation of decorin upon knockdown of periostin in BT-20 cells. Anti-periostin or anti-decorin immunoprecipitates from BT-20 cell lysates were subjected to SDS-PAGE, followed by immunoblot analysis with anti-decorin or anti-periostin, respectively. Input lysate was used as a positive control, and normal rabbit IgG was used as a negative control.Tissue lysates were immunoblotted with anti-decorin and anti-periostin antibodies. N, normal tissue; T, tumor tissue. These cropped blots are used in the main figure(Figure 3b).

Supplementary Figure S4

DLPPDTTLLDLQNNK [Lys (12C6, 14N2); 848.670 m/z (Q1), 957.480 m/z (Q3)]
DLPPDTTLLDLQNNK [Lys (13C6, 15N2); 852.670 m/z (Q1), 965.480 m/z (Q3)]
DLPPDTTLLDLQNNK [Lys (12C6, 14N2); 848.670 m/z (Q1), 957.480 m/z (Q3)]

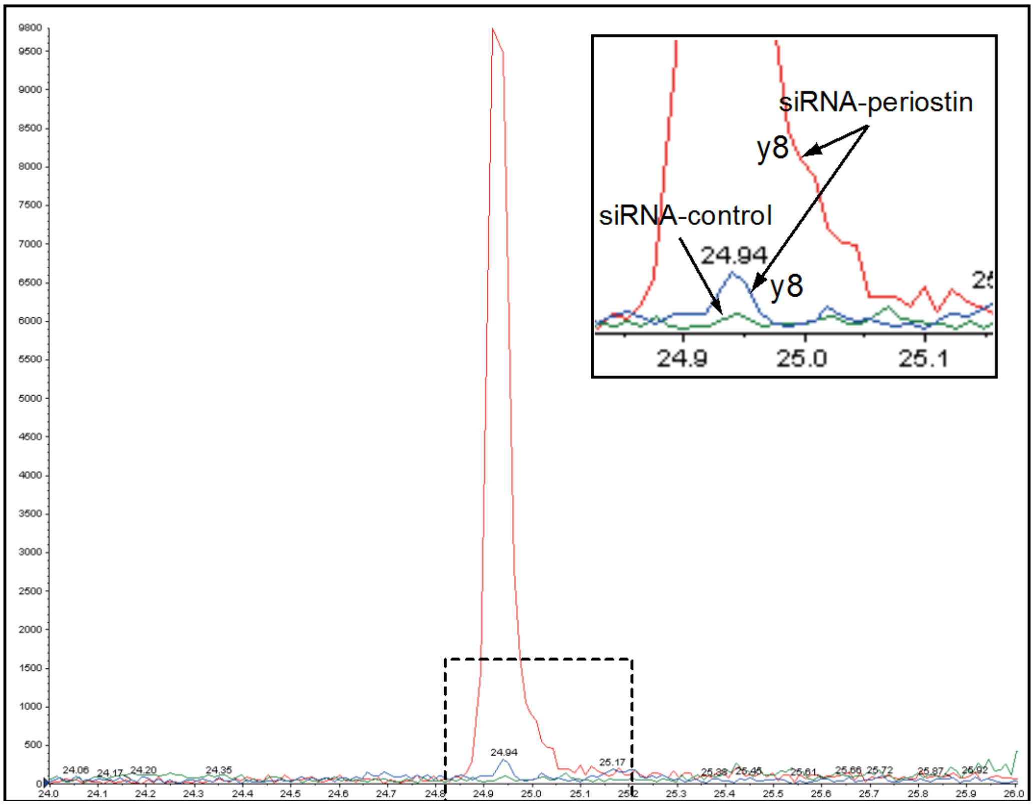

Supplementary Figure S4. MRM chromatograms for DLPPDTTLLDLQNNK fragments and their standard (AQUA: red line) analogues. MRM chromatograms for VSPGAFTPLVK was shown in Figure 4a. The peptides in culture medium from siRNA-periostin–treated cells (blue line) or siRNA-control–treated cells (green line) were analyzed using the MRM method. The doubly charged precursor mass was chosen as the Q1 mass, and the y8 fragment ion was chosen as the Q3 mass. Insets contain magnified views.

Supplementary Figure S5

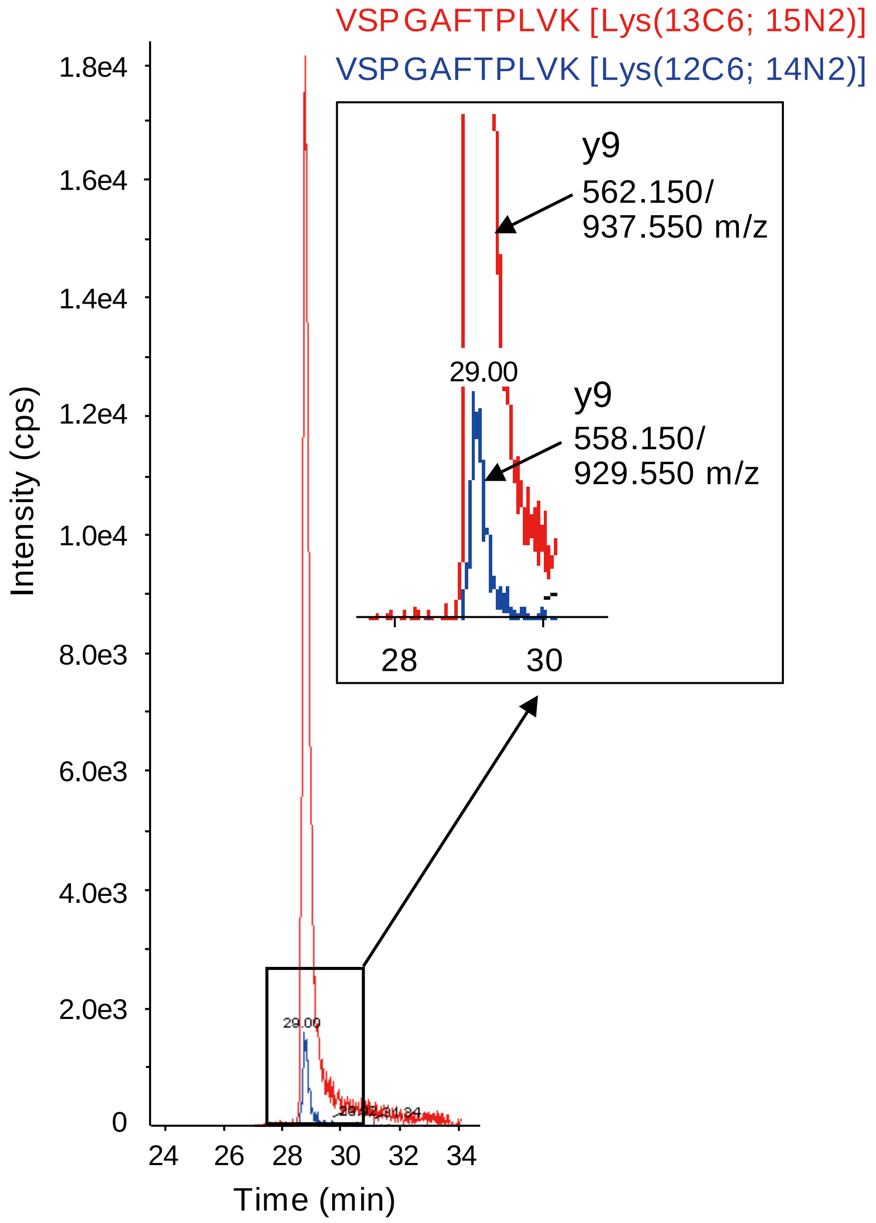

Supplementary Figure S5. MRM chromatograms for VSPGAFTPLVK fragments and their standard (AQUA) analogues. Two MRM transitions for the endogenous (blue line) and standard (red line) peptides were monitored(Figure 4b). For VSPGAFTPLVK, the doubly charged precursor mass was chosen as the Q1 mass, and the y9 fragment ions were chosen as Q3 mass. Insets contain magnified views. MRM-triggered MS/MS product ion spectra obtained by nanoflow LC/MS/MS, comparing normal tissue digested with trypsin (Firgure 4c; upper) with decorin secreted from decorin-overexpressing MDA-MB-231 cells (Figure 4c; lower). The spectrum of the peptide clearly shows y-ion fragments.

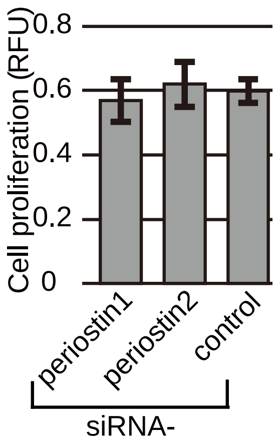

Supplementary Figure S6. Proliferation of BT-20 cells treated with siRNA-periostin or siRNA-control. Cell number was measured using water-soluble tetrazolium-1 (WST-1) assay. Relative fluorescence units (RFU) indicate the relative amount of proliferation. Column graphs show the means ± SEM of results from six samples.

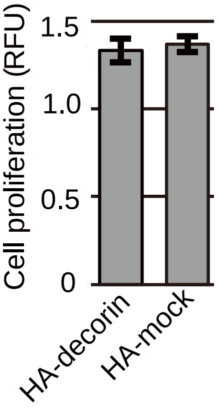

Supplementary Figure S7. Proliferation of MDA-MB-231 transiently expressing HA-decorin or HA-mock in MDA-MB-231 cells was measured using the WST-1 assay. Column graphs show means ± SEM of results from seven samples.

Supplementary Table S1

Accession #	Name	extracellular region	Species	Unused	% Cov	Peptides (95%)	117:114	Case 1						Case2						Case3					
								10mM	25mM	50mM	100mM	175mM	350mM	10mM	25mM	50mM	100mM	175mM	350mM	10mM	25mM	50mM	100mM	175mM	350mM
Up-regulataed proteins at tumor tissue																									
sp|Q15063|POSTN_HUMAN	Periostin OS=Homo sapiens GN=POSTN PE=1 SV=2	○	HUMAN	33.04	61.8	22	11.695		○	○	●	○	○	○	○	○	○	○	○			○	○	○	○
sp|P13611|CSPG2_HUMAN	Versican core protein OS=Homo sapiens GN=VCAN PE=1 SV=3	○	HUMAN	13.66	14.9	7	10.0925			○	●				○	○	○	○				○	○	○	
sp|P00738|HPT_HUMAN	Haptoglobin OS=Homo sapiens GN=HP PE=1 SV=1		HUMAN	16.34	42.4	9	7.7268		○	○	●	○	○								○				
sp|P02787|TRFE_HUMAN	Serotransferrin OS=Homo sapiens GN=TF PE=1 SV=3		HUMAN	60.46	63.2	33	8.7096		○	○	●	○	○												
sp|P22676|CALB2_HUMAN	Calretinin OS=Homo sapiens GN=CALB2 PE=1 SV=2		HUMAN	5.22	22.5	3	15.2757			○	●	○	○												
sp|P02647|APOA1_HUMAN	Apolipoprotein A-I OS=Homo sapiens GN=APOA1 PE=1 SV=1	○	HUMAN	21.66	65.5	12	4.0926	○		○	●	○													
sp|P11142|HSP7C_HUMAN	Heat shock cognate 71 kDa protein OS=Homo sapiens GN=HSPA8 PE=1 SV=1		HUMAN	8.82	22.1	6	3.8726															●	○		○
sp|P24821|TENA_HUMAN	Tenascin OS=Homo sapiens GN=TNC PE=1 SV=2	○	HUMAN	2	8.1	2	13.6773				○	○						●							
sp|P00450|CERU_HUMAN	Ceruloplasmin OS=Homo sapiens GN=CP PE=1 SV=1	○	HUMAN	14.49	30.2	10	3.8371				●	○	○												
sp|P35443|TSP4_HUMAN	Thrombospondin-4 OS=Homo sapiens GN=THBS4 PE=1 SV=2	○	HUMAN	4.07	17.7	3	29.6483		○	○	●														
sp|P08758|ANXA5_HUMAN	Annexin A5 OS=Homo sapiens GN=ANXA5 PE=1 SV=2		HUMAN	7.53	24.4	4	6.2517							○			●						○		
sp|P02765|FETUA_HUMAN	Alpha-2-HS-glycoprotein OS=Homo sapiens GN=AHSG PE=1 SV=1	○	HUMAN	6.02	33	2	4.0926			○		●								○					
sp|P02774|VTDB_HUMAN	Vitamin D-binding protein OS=Homo sapiens GN=GC PE=1 SV=1	○	HUMAN	8.88	35.7	5	8.7902				○	●	○												
sp|P02763|A1AG1_HUMAN	Alpha-1-acid glycoprotein 1 OS=Homo sapiens GN=ORM1 PE=1 SV=1		HUMAN	7.54	48.8	4	4.1687				○	●													
sp|P13639|EF2_HUMAN	Elongation factor 2 OS=Homo sapiens GN=EEF2 PE=1 SV=4		HUMAN	6.55	17.4	3	6.6681															●		○	
sp|P21333|FLNA_HUMAN	Filamin-A OS=Homo sapiens GN=FLNA PE=1 SV=4		HUMAN	36.52	25.7	18	3.8371															●			○
sp|P04406|G3P_HUMAN	Glyceraldehyde-3-phosphate dehydrogenase OS=Homo sapiens GN=GAPDH PE=1 SV=3		HUMAN	7.43	32.2	4	4.8306															●		○	
sp|P07737|PROF1_HUMAN	Profilin-1 OS=Homo sapiens GN=PFN1 PE=1 SV=2		HUMAN	7.22	55	5	3.9811									○						●			
sp|P11021|GRP78_HUMAN	78 kDa glucose-regulated protein OS=Homo sapiens GN=HSPA5 PE=1 SV=2		HUMAN	15.1	24.3	9	5.7016								○							●			
sp|P12814|ACTN1_HUMAN	Alpha-actinin-1 OS=Homo sapiens GN=ACTN1 PE=1 SV=2	○	HUMAN	8	17	4	6.4269										○	●							
sp|P01860|IGHG3_HUMAN	Ig gamma-3 chain C region OS=Homo sapiens GN=IGHG3 PE=1 SV=2	○	HUMAN	4.65	36.1	2	7.4473				○	●													
sp|Q6UXB8|PI16_HUMAN	Peptidase inhibitor 16 OS=Homo sapiens GN=PI16 PE=1 SV=1	○	HUMAN	2.06	14.9	1	11.3763																		●
sp|P62158|CALM_HUMAN	Calmodulin OS=Homo sapiens GN=CALM1 PE=1 SV=2		HUMAN	4	36.9	3	3.6983											●							
sp|P19012|K1C15_HUMAN	Keratin, type I cytoskeletal 15 OS=Homo sapiens GN=KRT15 PE=1 SV=2		HUMAN	8.05	27.6	5	10										●								
sp|O43707|ACTN4_HUMAN	Alpha-actinin-4 OS=Homo sapiens GN=ACTN4 PE=1 SV=2	○	HUMAN	7.57	25.3	3	5.2									●									
sp|P06733|ENOA_HUMAN	Alpha-enolase OS=Homo sapiens GN=ENO1 PE=1 SV=2		HUMAN	11.86	43.8	6	3.767									●									
sp|P08133|ANXA6_HUMAN	Annexin A6 OS=Homo sapiens GN=ANXA6 PE=1 SV=3		HUMAN	9.71	25.4	5	3.9811																	●	
sp|P02679|FIBG_HUMAN	Fibrinogen gamma chain OS=Homo sapiens GN=FGG PE=1 SV=3		HUMAN	5.13	27.2	2	8.091																	●	
sp|P05455|LA_HUMAN	Lupus La protein OS=Homo sapiens GN=SSB PE=1 SV=2		HUMAN	5.14	34.1	3	6.6681																	●	
sp|P55072|TERA_HUMAN	Transitional endoplasmic reticulum ATPase OS=Homo sapiens GN=VCP PE=1 SV=4		HUMAN	3.58	22.7	2	8.9536																	●	
sp|P07437|TBB5_HUMAN	Tubulin beta chain OS=Homo sapiens GN=TUBB PE=1 SV=2		HUMAN	10.47	41.9	5	5.1051																●		
sp|P12956|KU70_HUMAN	ATP-dependent DNA helicase 2 subunit 1 OS=Homo sapiens GN=XRCC6 PE=1 SV=2		HUMAN	1.37	14	1	3.3113															●			
sp|P23528|COF1_HUMAN	Cofilin-1 OS=Homo sapiens GN=CFL1 PE=1 SV=3		HUMAN	4.03	68.7	2	6.1376															●			
sp|P08238|HS90B_HUMAN	Heat shock protein HSP 90-beta OS=Homo sapiens GN=HSP90AB1 PE=1 SV=4		HUMAN	12.04	20.7	7	4.7863															●			
sp|P05783|K1C18_HUMAN	Keratin, type I cytoskeletal 18 OS=Homo sapiens GN=KRT18 PE=1 SV=2		HUMAN	12.29	36.3	6	4.3652															●			
sp|P07237|PDIA1_HUMAN	Protein disulfide-isomerase OS=Homo sapiens GN=P4HB PE=1 SV=3		HUMAN	4.46	23.2	2	3.5645															●			
sp|Q9BQE3|TBA1C_HUMAN	Tubulin alpha-1C chain OS=Homo sapiens GN=TUBA1C PE=1 SV=1		HUMAN	8.15	34.1	7	3.5975															●			
sp|Q16658|FSCN1_HUMAN	Fascin OS=Homo sapiens GN=FSCN1 PE=1 SV=3		HUMAN	4.28	33.3	2	7.4473														●				
sp|Q15365|PCBP1_HUMAN	Poly(rC)-binding protein 1 OS=Homo sapiens GN=PCBP1 PE=1 SV=2		HUMAN	3.4	14.9	3	10.0925														●				
sp|P34932|HSP74_HUMAN	Heat shock 70 kDa protein 4 OS=Homo sapiens GN=HSPA4 PE=1 SV=4		HUMAN	2	25.8	1	3.4995												●						
sp|P27797|CALR_HUMAN	Calreticulin OS=Homo sapiens GN=CALR PE=1 SV=1		HUMAN	2.38	13.2	1	4.1687										●								
sp|P18669|PGAM1_HUMAN	Phosphoglycerate mutase 1 OS=Homo sapiens GN=PGAM1 PE=1 SV=2		HUMAN	4	33.9	3	6.9823										●								
sp|Q71U36|TBA1A_HUMAN	Tubulin alpha-1A chain OS=Homo sapiens GN=TUBA1A PE=1 SV=1		HUMAN	11.98	37	5	4.7424										●								
sp|P02652|APOA2_HUMAN	Apolipoprotein A-II OS=Homo sapiens GN=APOA2 PE=1 SV=1	○	HUMAN	2.59	80	3	3.5975					●													
sp|P01009|A1AT_HUMAN	Alpha-1-antitrypsin OS=Homo sapiens GN=SERPINA1 PE=1 SV=3		HUMAN	24.99	63.9	12	3.02				●														
sp|P02675|FIBB_HUMAN	Fibrinogen beta chain OS=Homo sapiens GN=FGB PE=1 SV=2		HUMAN	11.75	36.9	4	4.6989																		●
sp|P68366|TBA4A_HUMAN	Tubulin alpha-4A chain OS=Homo sapiens GN=TUBA4A PE=1 SV=1		HUMAN	9.06	38.4	5	8.5507																	●	
sp|P04264|K2C1_HUMAN	Keratin, type II cytoskeletal 1 OS=Homo sapiens GN=KRT1 PE=1 SV=5		HUMAN	15.89	43.3	9	7.4473															●			
sp|P01871|IGHM_HUMAN	Ig mu chain C region OS=Homo sapiens GN=IGHM PE=1 SV=3	○	HUMAN	2	17.9	1	12.0226														●				
sp|Q07021|C1QBP_HUMAN	Complement component 1 Q subcomponent-binding protein, mitochondrial OS=Homo sapiens GN=C1QBP PE=1 SV=1		HUMAN	2	10.6	1	4.8306												●						
sp|P18206|VINC_HUMAN	Vinculin OS=Homo sapiens GN=VCL PE=1 SV=4		HUMAN	2.01	21.6	1	4.3251								●										
sp|P52209|6PGD_HUMAN	6-phosphogluconate dehydrogenase, decarboxylating OS=Homo sapiens GN=PGD PE=1 SV=3		HUMAN	2	23.4	1	5.445			●															
Up-regulataed proteins at normal tissue																									
sp|P68871|HBB_HUMAN	Hemoglobin subunit beta OS=Homo sapiens GN=HBB PE=1 SV=2		HUMAN	24.06	74.8	20	0.0637		○						○	○	○	○	○		○	○	○	○	●
sp|P20774|MIME_HUMAN	Mimecan OS=Homo sapiens GN=OGN PE=1 SV=1	○	HUMAN	6.6	36.9	5	0.0263				○	○	●		○			○	○			○	○	○	○
sp|P69905|HBA_HUMAN	Hemoglobin subunit alpha OS=Homo sapiens GN=HBA1 PE=1 SV=2		HUMAN	35.26	83.1	24	0.0698						○			○	○	○	○			○	○	○	●
sp|P08727|K1C19_HUMAN	Keratin, type I cytoskeletal 19 OS=Homo sapiens GN=KRT19 PE=1 SV=3		HUMAN	15.14	47.5	8	0.0334			○	○		○		○	○	●					○		○	
sp|P07585|PGS2_HUMAN	Decorin OS=Homo sapiens GN=DCN PE=1 SV=1	○	HUMAN	10.06	39.8	5	0.2109			○					○	○	○					○	●	○	
sp|P25311|ZA2G_HUMAN	Zinc-alpha-2-glycoprotein OS=Homo sapiens GN=AZGP1 PE=1 SV=1	○	HUMAN	20.6	74.9	12	0.0283		○	○	○	○	○	○	○	○	○	●	○						
sp|P12273|PIP_HUMAN	Prolactin-inducible protein OS=Homo sapiens GN=PIP PE=1 SV=1	○	HUMAN	14.77	78.8	8	0.0163		○	○	○			○	○	○	●	○	○						
sp|P02042|HBD_HUMAN	Hemoglobin subunit delta OS=Homo sapiens GN=HBD PE=1 SV=2		HUMAN	13.02	85	14	0.1306									○	○		○		○	○	○	●	
sp|P51884|LUM_HUMAN	Lumican OS=Homo sapiens GN=LUM PE=1 SV=2	○	HUMAN	14.93	50.3	9	0.1213															○	○	●	○
sp|O95994|AGR2_HUMAN	Anterior gradient protein 2 homolog OS=Homo sapiens GN=AGR2 PE=1 SV=1	○	HUMAN	4.05	44.6	2	0.0705			○	●	○	○												
sp|P01876|IGHA1_HUMAN	Ig alpha-1 chain C region OS=Homo sapiens GN=IGHA1 PE=1 SV=2		HUMAN	10.01	32.3	4	0.0575									●	○		○				○		
sp|P04264|K2C1_HUMAN	Keratin, type II cytoskeletal 1 OS=Homo sapiens GN=KRT1 PE=1 SV=5		HUMAN	5.33	27.3	4	0.0637								○	●	○		○						
sp|P08670|VIME_HUMAN	Vimentin OS=Homo sapiens GN=VIM PE=1 SV=4	○	HUMAN	15.98	46.4	8	0.1202			○	○	●	○												
sp|P08729|K2C7_HUMAN	Keratin, type II cytoskeletal 7 OS=Homo sapiens GN=KRT7 PE=1 SV=3		HUMAN	3.42	46.3	3	0.2377			○	●	○													
sp|P06703|S10A6_HUMAN	Protein S100-A6 OS=Homo sapiens GN=S100A6 PE=1 SV=1		HUMAN	3.93	30	2	0.1057															○	●		
sp|P01834|IGKC_HUMAN	Ig kappa chain C region OS=Homo sapiens GN=IGKC PE=1 SV=1	○	HUMAN	6.8	61.3	4	0.2089										●	○							
sp|P02652|APOA2_HUMAN	Apolipoprotein A-II OS=Homo sapiens GN=APOA2 PE=1 SV=1	○	HUMAN	3.75	94	4	0.2938											○						●	
sp|P00915|CAH1_HUMAN	Carbonic anhydrase 1 OS=Homo sapiens GN=CA1 PE=1 SV=2		HUMAN	4.02	28	4	0.2399															●	○		
sp|Q6NZI2|PTRF_HUMAN	Polymerase I and transcript release factor OS=Homo sapiens GN=PTRF PE=1 SV=1		HUMAN	2	12.6	2	0.0692														●	○			
sp|P32119|PRDX2_HUMAN	Peroxiredoxin-2 OS=Homo sapiens GN=PRDX2 PE=1 SV=5		HUMAN	6.49	35.4	5	0.2831															○	●		
sp|P08123|CO1A2_HUMAN	Collagen alpha-2(I) chain OS=Homo sapiens GN=COL1A2 PE=1 SV=6	○	HUMAN	16.67	41	9	0.0887														○	●			
sp|P05787|K2C8_HUMAN	Keratin, type II cytoskeletal 8 OS=Homo sapiens GN=KRT8 PE=1 SV=7		HUMAN	6.39	42.9	3	0.0461				●	○													
sp|P01023|A2MG_HUMAN	Alpha-2-macroglobulin OS=Homo sapiens GN=A2M PE=1 SV=1		HUMAN	13.58	31.1	5	0.1127			●	○														
sp|Q09666|AHNK_HUMAN	Neuroblast differentiation-associated protein AHNAK OS=Homo sapiens GN=AHNAK PE=1 SV=2		HUMAN	6.55	32.1	3	0.2148																		●
sp|P07339|CATD_HUMAN	Cathepsin D OS=Homo sapiens GN=CTSD PE=1 SV=1	○	HUMAN	7.7	24.5	5	0.1406																●		
sp|P69892|HBG2_HUMAN	Hemoglobin subunit gamma-2 OS=Homo sapiens GN=HBG2 PE=1 SV=2		HUMAN	2.5	47.6	1	0.1306															●			
sp|P13667|PDIA4_HUMAN	Protein disulfide-isomerase A4 OS=Homo sapiens GN=PDIA4 PE=1 SV=2		HUMAN	4.01	12.9	2	0.0863															●			
sp|P15090|FABP4_HUMAN	Fatty acid-binding protein, adipocyte OS=Homo sapiens GN=FABP4 PE=1 SV=3		HUMAN	2.2	31.1	1	0.0802										●								
sp|P02768|ALBU_HUMAN	Serum albumin OS=Homo sapiens GN=ALB PE=1 SV=2	○	HUMAN	36.98	58.6	20	0.2938								●										
sp|P08758|ANXA5_HUMAN	Annexin A5 OS=Homo sapiens GN=ANXA5 PE=1 SV=2		HUMAN	10.23	49.7	6	0.2884						●												
sp|P07355|ANXA2_HUMAN	Annexin A2 OS=Homo sapiens GN=ANXA2 PE=1 SV=2	○	HUMAN	5.22	41.6	2	0.2249					●													
sp|Q9NZT1|CALL5_HUMAN	Calmodulin-like protein 5 OS=Homo sapiens GN=CALML5 PE=1 SV=2		HUMAN	2	23.3	1	0.2582			●															
sp|P23297|S10A1_HUMAN	Protein S100-A1 OS=Homo sapiens GN=S100A1 PE=1 SV=2		HUMAN	1.3	16	1	0.1294																●		
sp|Q14956|GPNMB_HUMAN	Transmembrane glycoprotein NMB OS=Homo sapiens GN=GPNMB PE=1 SV=2		HUMAN	2	15.2	1	0.2489																●		
sp|P02452|CO1A1_HUMAN	Collagen alpha-1(I) chain OS=Homo sapiens GN=COL1A1 PE=1 SV=4	○	HUMAN	27.06	45.7	15	0.055															●			
sp|P07108|ACBP_HUMAN	Acyl-CoA-binding protein OS=Homo sapiens GN=DBI PE=1 SV=2		HUMAN	2	37.9	2	0.036														●				
sp|P30041|PRDX6_HUMAN	Peroxiredoxin-6 OS=Homo sapiens GN=PRDX6 PE=1 SV=3		HUMAN	3.16	58.9	1	0.138												●						
sp|P01011|AACT_HUMAN	Alpha-1-antichymotrypsin OS=Homo sapiens GN=SERPINA3 PE=1 SV=2		HUMAN	7.32	21.8	5	0.1803										●								
sp|P02675|FIBB_HUMAN	Fibrinogen beta chain OS=Homo sapiens GN=FGB PE=1 SV=2		HUMAN	7.5	27.9	4	0.2399										●								
sp|P01842|LAC_HUMAN	Ig lambda chain C regions OS=Homo sapiens GN=IGLC1 PE=1 SV=1	○	HUMAN	4	56.2	2	0.2512										●								
sp|Q6UXB8|PI16_HUMAN	Peptidase inhibitor 16 OS=Homo sapiens GN=PI16 PE=1 SV=1	○	HUMAN	2	15.1	2	0.2109						●												
sp|Q6W4X9|MUC6_HUMAN	Mucin-6 OS=Homo sapiens GN=MUC6 PE=1 SV=2	○	HUMAN	6.62	11	3	0.0802			●															
sp|Q9Y247|FA50B_HUMAN	Protein FAM50B OS=Homo sapiens GN=FAM50B PE=2 SV=1		HUMAN	0.15	27.1	0	0.0296																●		
sp|Q53EL6|PDCD4_HUMAN	Programmed cell death protein 4 OS=Homo sapiens GN=PDCD4 PE=1 SV=1		HUMAN	1.3	28.1	1	0.0685												●						
sp|P09972|ALDOC_HUMAN	Fructose-bisphosphate aldolase C OS=Homo sapiens GN=ALDOC PE=1 SV=2		HUMAN	2.17	29.4	1	0.3162									●									
sp|P07237|PDIA1_HUMAN	Protein disulfide-isomerase OS=Homo sapiens GN=P4HB PE=1 SV=3		HUMAN	5.59	42.9	3	0.2858						●												
sp|P21266|GSTM3_HUMAN	Glutathione S-transferase Mu 3 OS=Homo sapiens GN=GSTM3 PE=1 SV=3		HUMAN	1.33	28.4	1	0.2333					●													
sp|Q07654|TFF3_HUMAN	Trefoil factor 3 OS=Homo sapiens GN=TFF3 PE=1 SV=1	○	HUMAN	0.22	35	0	0.1486					●													
sp|P08779|K1C16_HUMAN	Keratin, type I cytoskeletal 16 OS=Homo sapiens GN=KRT16 PE=1 SV=4		HUMAN	2.23	31.7	0	0.1614				●														

Supplementary Table S1. Clinical characteristics of phyllodes tumors and fibroadenomas. Open and closed circles show results that were detected more than once in three measurements. Unused, 95% confidence peptides, and 114 vs. 117 ratio were shown from data marked by closed circles.

Supplementary Table S2

IP:decorin

			Unused	% Cov	Name	Peptides(95%)
1	case1	Normal	9.67	48.5	Decorin	4
		Tumor	4.06	48.2	Decorin	1
	case2	Normal	14.06	53.8	Decorin	7
		Tumor	4.42	59.3	Decorin	2
	case3	Normal	18.61	61	Decorin	10
		Tumor	14.1	50.1	Decorin	7
2	case1	Normal	3.65	28.6	Periostin	1
			2.55	53.2	Decorin	1
		Tumor	11.45	43.4	Periostin	5
			7.19	28.1	Decorin	3
	case2	Normal	20.75	46.5	Decorin	13
		Tumor	4.06	32	Decorin	2
	case3	Normal	11.74	56.8	Decorin	5
			2.06	39.1	Dermcidin	1
		Tumor	1.7	13.6	Hornerin	1
3	case1	Normal	2	35.1	60 kDa heat shock protein, mitochondrial	1
		Tumor	10.39	30.1	Alpha-1-antitrypsin	6
	case2	Tumor	2.21	22	Alpha-1-antitrypsin	1
			2	22.5	60 kDa heat shock protein, mitochondrial	2
	case3	Tumor	2.74	32.3	60 kDa heat shock protein, mitochondrial	1
			2.46	31.3	Alpha-1-antitrypsin	1
4	case1	Normal	11.91	43.2	Actin, cytoplasmic 1	6
			8.26	65.2	Vimentin	3
			0.35	23.9	Beta-actin-like protein 2	1
		Tumor	8.98	40	Actin, cytoplasmic 1	4
	case2	Normal	15.01	50.1	Actin, cytoplasmic 2	5
			3.34	40.3	Vimentin	2
			2.53	36.2	Decorin	1
		Tumor	14.93	49.1	Actin, cytoplasmic 2	8
			2	49.1	Actin, cytoplasmic 1	8
	case3	Normal	11.1	42.9	Actin, cytoplasmic 1	4
		Tumor	16.13	56.3	Actin, cytoplasmic 1	8
			0.64	56	Actin, cytoplasmic 2	7
			0.46	38.8	Beta-actin-like protein 2	2
			0.18	54.1	Actin, aortic smooth muscle	3

IP:periostin

			Unused	% Cov	Name	Peptides(95%)
5	case1	Normal	1.3	11.3	Voltage-dependent L-type calcium channel subunit alpha-1C	1
		Tumor	2	12.8	Alpha-2-macroglobulin	1
6	case1	Tumor	4.26	31.8	Decorin	2
	case2	Normal	28.51	73.8	Decorin	16
		Tumor	3.54	35.4	Decorin	2
	case3	Normal	2.42	27.6	Decorin	1
		Tumor	13.7	39.6	Decorin	5
7	case1	Normal	17.74	46.2	Decorin	8
			14.54	42.1	Periostin	4
		Tumor	25.97	55.1	Periostin	10
			4.15	34.3	Decorin	2
	case2	Normal	41.92	53.2	Decorin	23
			3.55	22.4	Periostin	1
		Tumor	14.93	37.3	Periostin	8
			3.79	36.2	Decorin	2
			2.38	43.6	Dermcidin	1
	case3	Normal	3.56	33.7	Decorin	1
		Tumor	7.84	37.9	Decorin	3
			5.42	33.1	Periostin	2
8	case1	Normal	1.47	18.6	Lumican	1
			1.36	30	Putative V-set and immunoglobulin domain-containing-like protein ENSP00000303034	1
		Tumor	10.72	45.7	Alpha-1-antitrypsin	3
	case2	Normal	2.07	28.7	Alpha-1-antitrypsin	1
	case3	Tumor	2	14.8	60 kDa heat shock protein, mitochondrial	1
9	case1	Normal	1.3	12.2	Nuclear pore membrane glycoprotein 210	1

Supplementary Table S2. List of decorin- or periostin-binding target proteins from Figure 3e and f.

cell	medium
BT-20	Eagle's Minimum Essential Medium with L-Glutamine (ATCC, Manassas, VA, USA) supplemented with 10% fetal bovine serum
T-47D	RPMI-1640 with High Glucose, L-glutamine, and HEPES (ATCC) supplemented with 10% fetal bovine serum and insulin
MDA-MB-231	DMEM/F12 (1:1) with L-Glutamine (Life Technologies, Grand Island, NY, USA) supplemented with 10% fetal bovine serum and HEPES
MCF7	Eagle's Minimum Essential Medium with 10% fetal bovine serum and 0.01 mg/ml human recombinant insulin
HeLa S3	DMEM with 10% fetal bovine serum, $NaHCO_3$, and L-glutamine

Supplementary Table S3. List of the mediums for each cell line.

Application	antibody	species	source
Immunoblotting	decorin	mouse	Santa Cruz Biotechnology, Dallas, TX, USA
	periostin	rabbit	Abcam, Cambridge, UK
	E-cadherin	mouse	Takara, Otsu, Japan
	β-actin	mouse	Sigma-Aldrich, St. LouisMO, USA
Immunoprecipitation	decorin	rabbit	Bioss, Woburn, MA, USA
	periostin	rabbit	Santa Cruz Biotechnology, Dallas, TX, USA
Immunohistochemical staining	decorin	mouse	Abcam, Cambridge, UK
	periostin	rabbit	Abcam, Cambridge, UK
Immunofluorescence	decorin	mouse	Abcam, Cambridge, UK
	periostin	rabbit	GeneTex, Irvine, CA, USA

Supplementary Table S4. List of antibodies.

Supplementary Method

Immunoblotting analysis

Tissue and cell lysates were subjected to SDS-PAGE and transferred electrophoretically onto Immobilon-P transfer membrane (EMD Millipore, Billerica, MA, USA). The membrane was incubated in SuperBlock Blocking Buffer (Thermo Scientific, Waltham, MA, USA) at room temperature (RT) for 1 h, stained with primary antibodies for 2 h at RT, and incubated for 1 h at RT with secondary antibodies coupled to horseradish peroxidase (ECL anti-mouse or anti-rabbit IgG; Amersham/GE Healthcare, Buckinghamshire, UK). The blots were developed using the SuperSignal enhanced chemiluminescence reagent (Pierce, Rockford, IL, USA) and exposed to Kodak X-OMAT film. All antibodies are listed in Supplementary Table S4. As needed, protein bands were quantitated using the ImageJ software (version 1.46r, National Institutes of Health, Bethesda, MD, USA).

Immunoprecipitation

Tissue and cell lysates were immunoprecipitated for 30 min at 4° C with 5 μg antibody, followed by incubation for 30 min at 4° C with Dynabeads–Protein G (Invitrogen, Carlsbad, CA, USA). Immune complexes were precipitated and washed. Immunoprecipitated proteins were loaded to onto e-PAGEL precast gels (ATTO, Tokyo, Japan) for mass spectrometry or subjected to standard SDS-PAGE for immunoblotting.

Knockdown of gene expression

siRNAs specifically targeting periostin and decorin were purchased from Dharmacon (Lafayette, CO, USA. For transfections, Lipofectamine RNAiMAX (Invitrogen) and 12 nM siRNA were mixed in 2 ml Opti-MEM (Life Technologies) and incubated for 20 minutes at RT; the mixture was then added onto 70% confluent cultures. After 72 h, RNA and protein were isolated.

Total RNA extraction and RT-PCR

Total RNA was extracted from the lysates BT-20 using the QuickPrep Micro mRNA Purification Kit (GE Healthcare, Little Chalfont, UK). Decorin full-length cDNA was generated from RNA by RT-PCR using the SuperScript III One-Step RT-PCR System with Platinum Taq (Life Technologies) and subcloned into the pME18S-HA-C vector in frame with the appropriate N-terminal tags. Clonal cell lines were obtained by transfection of 2 μg of plasmid DNA using the Lipofectamine Plus transfection reagent (Invitrogen). Reverse transcription was performed in a reaction volume of 60 ml containing 90 ng of total RNA and 2 ml of SuperScript III One-Step RT-PCR System with Platinum Taq. The conditions for polymerase chain reaction (PCR) were as follows: 94° C for 2 min; 40 cycles of 94° C for 15 s, 60° C for 30 s, and 68° C for 90 s; and a final cycle of 68° C for 5 min. The PCR products were electrophoresed on 1% agarose gels. The PCR primers for *DCN* were 5’-CCGGAATTCATGAAGGCCACTATCATCC-3’ and 5’-GCCGCTCGAGTTACTTATAGTTTCCGAG-3’.

Immunofluorescence

Cells were seeded onto chamber slides (Matsunami, Osaka, Japan) and fixed with 3.5% formaldehyde in PBS for 10 minutes on ice. Fixed cells were permeabilized with 50%, 75%, and 95% cold ethanol on ice for 5 minutes each. Fixed cells were blocked for 30 min, incubated with primary antibody for 1 h at RT, incubated with Alexa Fluor 488– or Alexa Fluor 594–conjugated secondary antibody (Molecular Probes, Life Technologies) for 30 min at 37° C, and then preserved in Vectashield (Vector Laboratories, Burlingame, CA, USA). DNA was stained with bisbenzimide (Hoechst 33258, Life Technologies). The samples were examined with an Olympus Power BX51 fluorescence microscope (Olympus, Tokyo, Japan). Confocal microscopy was performed using a Leica TS SP8, and the confocal images were analyzed using the Leica SP8 LAS AF Lite software (Leica, Solms, Germany).

Cell proliferation assay

Cells were seeded in 96-well flat-bottomed microtiter plates and cultured for 24 h, followed by incubation for 30 minutes with Cell Proliferation Reagent WST-1 (Roche). Cleavage of WST-1 to formazan by metabolically active cells was quantitated by scanning the plates at 450 nm and 650 nm (reference wavelength) in a microtiter plate reader. Viability of treated cells was normalized to that of untreated control cells.
